# Learning hybrid locomotion skills—Learn to exploit residual actions and modulate model-based gait control

**DOI:** 10.3389/frobt.2023.1004490

**Published:** 2023-04-10

**Authors:** Mohammadreza Kasaei, Miguel Abreu, Nuno Lau, Artur Pereira, Luis Paulo Reis, Zhibin Li

**Affiliations:** ^1^ School of Informatics, University of Edinburgh, Edinburgh, United Kingdom; ^2^ University of Porto, LIACC / LASI / FEUP, Artificial Intelligence and Computer Science Lab, Faculty of Engineering of the University of Porto, Porto, Portugal; ^3^ IEETA / LASI / DETI University of Aveiro, Aveiro, Portugal; ^4^ Department of Computer Science, University College London, London, United Kingdom

**Keywords:** learning motor skills, humanoid robot, learning residual actions, modulate gait generator, deep reinforcement learning (DRL)

## Abstract

This work has developed a hybrid framework that combines machine learning and control approaches for legged robots to achieve new capabilities of balancing against external perturbations. The framework embeds a kernel which is a model-based, full parametric closed-loop and analytical controller as the gait pattern generator. On top of that, a neural network with symmetric partial data augmentation learns to automatically adjust the parameters for the gait kernel, and also generate compensatory actions for all joints, thus significantly augmenting the stability under unexpected perturbations. Seven Neural Network policies with different configurations were optimized to validate the effectiveness and the combined use of the modulation of the kernel parameters and the compensation for the arms and legs using residual actions. The results validated that modulating kernel parameters alongside the residual actions have improved the stability significantly. Furthermore, The performance of the proposed framework was evaluated across a set of challenging simulated scenarios, and demonstrated considerable improvements compared to the baseline in recovering from large external forces (up to 118%). Besides, regarding measurement noise and model inaccuracies, the robustness of the proposed framework has been assessed through simulations, which demonstrated the robustness in the presence of these uncertainties. Furthermore, the trained policies were validated across a set of unseen scenarios and showed the generalization to dynamic walking.

## 1 Introduction

Legged robots are versatile on irregular grounds and can be used in a wide range of applications. Nevertheless, robust locomotion is a complex research that still needs investigation. The stability of movements is an essential requirement for a robot to act safely in a real environment. A general question is: despite the versatility of legged robots, why aren’t they as capable as us yet? This work aims to improve the stability of legged locomotion in order to increase its versatility.

To achieve the versatility as intended, we investigated the fundamental aspect of learning balance recovery strategies. Humans combine a set of strategies (e.g., moving arms, ankles, hips, taking a step, etc.) to regain balance after facing an external disturbance. They rely on past experiences to improve their methods. Moreover, we investigated existing biped robot locomotion frameworks. Despite their stability have been improved significantly but they are not stable and safe enough to be utilised in our daily-life environments. Several approaches for stabilising a biped robot have been proposed that can be categorised into three major categories. In the remainder of this section, these categories will be introduced and some recent works in each category will be briefly reviewed.

### 1.1 Model-based analytical approaches

The basic idea behind the approaches in this category is using a dynamics model of the robot and designing a set of controllers, e.g., force controller (Mason et al., 2016), hybrid position/force (Faraji et al., 2019), admittance controller (Caron, 2020), based on specific criteria to minimise the tracking error. The most widely used model in literature is the Linear Inverted Pendulum (LIP) which abstracts the overall dynamics of a robot as a single mass. It restricts the vertical movement of the mass to provide a linear model which yields a fast solution for real-time implementations. This model has been investigated and extended for decades to design and analyse legged robot locomotion (Takenaka et al., 2009; Englsberger et al., 2015).

The Divergent Component of Motion (DCM) concept has been proposed in (Takenaka et al., 2009) that splits the LIP’s dynamics into stable and unstable parts, such that controlling the unstable part is enough for keeping the stability. In (Englsberger et al., 2015), DCM has been extended to 3D and, several control approaches including classical feedback controllers (Morisawa et al., 2014), Linear Quadratic Regulator (LQR)-based methods (Faraji et al., 2019; Kasaei et al., 2019) and the Model Predictive Control (MPC) (Brasseur et al., 2015; Marcucci et al., 2017; Posa et al., 2017; Zhou et al., 2017; Kasaei et al., 2021) have been used to formulate biped locomotion frameworks. All of them have been trying to compensate the tracking error by using a combination of three strategies, which are: manipulating the Ground Reaction Force (GRF) and modifying the position and time of the next step. Recently, more efforts have been made to go beyond the simplified LIP assumptions (e.g., COM vertical motion and angular momentum) and to deal with more complex non-linearities of multi-body dynamics (Kajita et al., 2018; Seyde et al., 2018; Caron, 2020; Chatzinikolaidis et al., 2020).

Model-based approaches can provide a way to generate stable and efficient gaits for legged robots, as they can be used to find the optimal solution for a given set of constraints and objectives, such as contact models of soft grounds (Chatzinikolaidis et al., 2020). However, these methods can be computationally very expensive and may not be able to handle the complexity of real-world environments. Additionally, most of the model-based approaches (specifically the optimization-based approaches) require an accurate model of the robot dynamics, which can be difficult to obtain in practice. Inaccuracies in the model can lead to poor performance or instability. Moreover, model-based approaches often rely on making assumptions or simplifications in the problem formulation, which can lead to limitations in the quality or applicability of the solutions. Besides, the optimization problem needs to be formulated carefully, to take into account all the constraints that the robot needs to adhere to.

### 1.2 Machine learning approaches

The approaches in this category are designed to learn a feasible policy through interaction with the environment. Nowadays, Deep Reinforcement Learning (DRL) has shown its capability by solving complex locomotion and manipulation tasks, which are generally composed of high-dimensional continuous observation and action spaces (Gu et al., 2017; Abreu et al., 2019a).

One key benefit of DRL approaches is that they can handle high-dimensional, non-linear, and continuous state and action spaces, which can make them well-suited to complex problems such as robotics and control. Additionally, DRL approaches can learn from raw sensor data, without the need for hand-engineered features, which can make them more robust to changes in the environment. However, DRL approaches have some limitations as well. One limitation is that they can require a large amount of data and computational resources to train, especially for problems with high-dimensional state and action spaces. Additionally, DRL approaches can be sensitive to the choice of hyperparameters.

Data augmentation in DRL is widely used to improve the optimization performance but, in this work, we restrict the scope to symmetry oriented solutions. The process of generating symmetric data from actual samples is used to improve different robotic tasks (Lin et al., 2020), including dynamic walking of various humanoid models Abdolhosseini et al. (2019) and quadruped robots Mishra et al. (2019); Yang C. et al. (2020). Learning from scratch with DRL can achieve very efficient behaviours, even in asymmetrical configurations (Abreu et al., 2019b). However, if not regulated through model restrictions (e.g., symmetry, pattern generators), it can be challenging to produce human-like behaviours in a reasonable amount of time.

In the case of humanoid locomotion, DRL might be more appropriate due to the complexity of the problem, high-dimensional states, and non-linear dynamics. On the other hand, optimization-based approaches may be more appropriate if we have good model of the robot and a clear mathematical objective, and if the goal is to track a specific trajectory. It is worth noting that both approaches could be combined to benefit from the advantages of both methods. For example, model-based reinforcement learning, which combines elements of optimization-based and DRL methods, has been applied to a variety of robotic control problems, including legged locomotion Yang Y. et al. (2020).

Reservoir computing and liquid state machines (LSM) have been proposed as alternative approaches for motor skill learning in multi-legged robots. These techniques utilize a fixed, randomly generated network of neurons, known as a reservoir, to process input data and generate output. In Franco-Robles et al. (2020), a method that utilizes a LSM to compute movement profiles has been proposed and they used a set of numerical experiments to validated the performance of their method. The results showed that the gait of the bipedal robot is stable in terms of the zero moment point (ZMP) when using the movement profiles generated by the LSM approach.

### 1.3 Hybrid approaches: Combing analytical and learning

The approaches in this category are focused on combining the potential of both aforementioned categories which can allow to take advantage of the strengths of both methods. Model-based methods can provide a starting point for learning, which can then be refined through learning from experience. Additionally, learned models can be incorporated into the optimization process to improve its performance and speed. This integration can be useful for providing more robust, adaptive and efficient solutions for legged robot locomotion, especially when facing uncertainty or changing environments. In this type of frameworks, learning algorithms are combined with model-based gait pattern generators to predict the parameters and to learn residual dynamics or residual actions, which can lead to impressively accurate behaviours (Koryakovskiy et al., 2018; Ahn et al., 2020; Li et al., 2021; Krishna et al., 2022). These frameworks are generally composed of a set of layers that are connected together in hierarchical structures.

In (Yang et al., 2018) a hierarchical framework has been designed to ensure the stability of a humanoid robot by learning motor skills. Their framework is composed of two independent layers, the high-level layer generates a set of joint angles and the low-level layer translates those angles to joint torques using a set of PD controllers. Their reward function was composed of six distinct terms that were mostly related to the traditional push recovery strategies, and it was obtained by adding all terms together with different weights. A reinforcement learning based controller for robust parameterized locomotion control of bipedal robots has been proposed in (Li et al., 2021). Indeed, they used Hybrid Zero Dynamics (HZD) approach to generate a gait library consists of periodic joint trajectories that encode a locomotion pattern, then, augmented it with deep reinforcement learning to develop a versatile locomotion. In (Krishna et al., 2022), a control pipeline has been proposed that validates linear policies are good enough for generating robust bipedal walking even on challenging terrains. This pipeline is composed of a high-level trajectory modulator and a low-level controller. The former modulates the end-foot trajectories and the later is responsible for regulating torso and ankle orientation. The performance of this pipeline has been validated through a set of simulations and real robot experiments including walking on constant inclines, declines, varying inclines, sinusoidal terrains and stairs.

### 1.4 Overview of the proposed framework and contributions

This work focuses on bipedal locomotion and push recovery which is the most challenging in legged robots. Particularly, we aim to investigate the effectiveness of employing a learning algorithm to control and modulate a model-based control policy such as a gait pattern generator. Our contributions are as follows.

**FIGURE 1:**
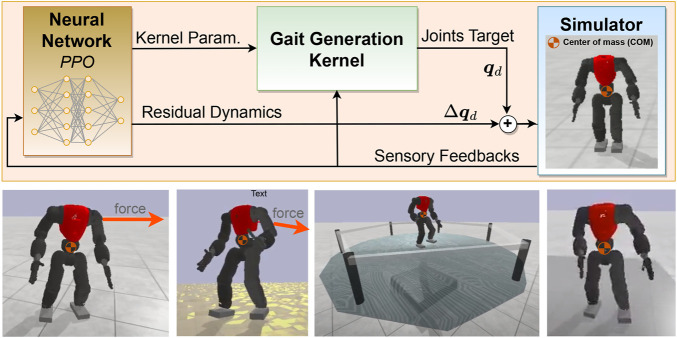
Overview of the proposed framework along with a set of snapshots of tests on different terrains: the gait generation kernel produces closed-loop locomotion, the neural network regulates the kernel’s parameters and generates compensatory actions.

• A hybrid locomotion framework (Section 2; Section 3). We developed a locomotion framework for humanoid robots that integrates both analytical control and machine learning. The kernel is formulated as a parametric model-based kernel to let the policy select the parameters alongside adding the residual actions (Overview in Figure 1).
• **Proposed motion symmetry to improve learning time and human-likeness (Section 3)**. We proposed a learning method where the data is only partially augmented, leveraging the symmetry to improve learning time and human-likeness without restricting asymmetric movements, thus widening the range of possible behaviours.
• **Benchmarking of the effectiveness of residual actions and modulation of the gait kernel (Section 5)**. Using the proposed framework, we optimized seven NN policies to investigate the effectiveness of adding residual actions to the arms and legs along with modulating the kernel parameters, and we compared the effectiveness of each configuration. We showed that modulating kernel parameters alongside adding residual actions leads to the most significant improvement.

The remainder of this paper is structured as follows. In Section 2, the architecture of our fully parametric kennel pattern generator will be presented and each module will be explained. Afterwards, in Section 3, our learning framework will be introduced and we will explain how we augmented this framework with the kernel pattern generator to regulate kernel parameters and to learn model-free skills (generating compensatory joint positions). In Section 4, a set of simulation scenarios will be designed to validate the performance of the proposed framework. Afterwards, in Section 5, a set of simulations will be conducted to provide assessments and analysis regarding overall performance, optimized policy behaviour, symmetry, and robustness. Finally, conclusions and future research are presented in Section 6.

## 2 Gait generation kernel

**FIGURE 2:**
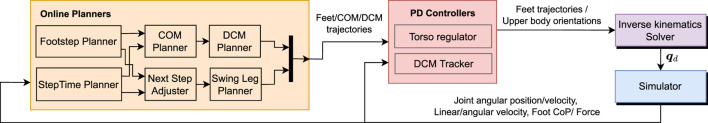
Overview of the proposed kernel gait generator. The online planners module generates a set of reference trajectories according to the input command and the states of the system. The PD controllers module is responsible for tracking the generated trajectories.

A fully parametric closed-loop gait generator serves as a kernel of the walking pattern (Figure 2). The gait generator is composed of two main modules: Online Planners and PD Controllers. Online Planners is responsible for generating the reference trajectories according to the stride’s parameters provided by the user, the robot’s state and the controllers’ output. PD Controllers regulates the upper body orientation and tracks the planned trajectories to generate closed-loop locomotion. The corresponding target joint positions are generated using Inverse Kinematics Solver, taking into account the kinematic feasibility. The target joint positions are fed to the Simulator for simulating the interaction of the robot with the environment and producing sensory data, as well as the global position and orientation of the robot.

### 2.1 Online planners

The Online Planners here is based on the work in (Kasaei et al., 2019), here we describe briefly the technical essentials. As shown in Figure 2, Online Planners is composed of a set of sub-planners which are solved separately and connected together hierarchically to reduce the complexity of the planning process. The planning process starts by generating a set of footsteps $\left(f_{i}={\left[f_{i_{x}}\;f_{i_{y}}\right]}^{⊤}\;i\in N\right)$ according to the input stride’s parameters and the current feet configuration. Then, the step time planner assigns a set of timestamps to the generated footstep according to the stride duration. Afterwards, to have a smooth trajectory during lifting and landing of the swing foot, a cubic spline is used to generate the swing leg trajectory based on the generated footsteps and a predefined swing height.

Accordingly, the COM planner generates the COM trajectory by solving LIP equation as a boundary value problem based on the generated footsteps. Then, the DCM trajectory can be obtained by substituting the generated COM and its time derivative into DCM equation ( $\zeta =c+\frac{\dot{c}}{\omega }$ , where ***ζ*** is DCM; ***c*** and $\dot{c}$ represent the COM and its time derivative, respectively, $\omega =\sqrt{\frac{g}{c_{z}}}$ is the natural frequency of the pendulum, where *g* is the gravity constant and *c*_*z*_ represents the height of the COM). This trajectory will be fed into PD Controllers to generate closed-loop locomotion. More detail can be found in our previous work (Kasaei et al., 2019).

In some situations, such as when the robot is being pushed severely, the DCM tracker cannot track the reference because of the controllers’ output saturation. In such conditions, humans adjust the next step time and location, in addition to the COM’s height. Due to the observability of DCM at each control cycle, the position of the next step can be determined by solving DCM equation as an initial value problem:

$$f_{i+1}=f_{i}+\left(\zeta_{t}-f_{i}\right)e^{\omega \left(T-t\right)},$$ (1)

where *f*_*i*_, *f*_*i*+1_ are the current and next support foot positions and *t*, *T* denote the time and stride duration, respectively.

It should be noted that adjusting the next stride time as well as the height of the COM is not straightforward due to non-linearities. Finding optimal or near optimal values for these parameters using DRL is a desirable solution, not only due to its convergence properties, but also because it allows us to find a more complete overall strategy by combining the stride time and COM height with residual adjustments.

### 2.2 Regulating the upper body orientation

The upper body of a humanoid is generally composed of several joints. While the robot is walking, their motions and vibrations generate angular momentum around the COM. To cancel the effects of this momentum, we designed a PD controller (***u*_Φ_**) based on the inertial sensor values that are mounted on the robot’s torso:

$$u_{\Phi }=-K_{\Phi }\left(\Phi_{d}-\Phi \right),$$ (2)

where $\Phi ={\left[\Phi_{\mathrm{roll}}\;{\dot{\Phi }}_{\mathrm{roll}}\;\Phi_{\mathrm{pitch}}\;{\dot{\Phi }}_{\mathrm{pitch}}\right]}^{⊤}$ represents the state of the torso and **Φ**_*d*_ denotes the desired state of the torso and ***K*_Φ_** is the controller gains.

### 2.3 DCM tracker

According to the LIP and DCM, the overall dynamics of a humanoid robot can be can be represented by a linear state space system as follows:

$$\frac{d}{dt}\left[\begin{array}{c} c\\ \zeta \end{array}\right]=\left[\begin{array}{cc} -\omega I_{2} & \omega I_{2}\\ 0I_{2} & \omega I_{2} \end{array}\right]\left[\begin{array}{c} c\\ \zeta \end{array}\right]+\left[\begin{array}{c} 0_{2\times 1}\\ -\Omega \end{array}\right]p,$$ (3)

where ***I***_2_ is an identity matrix of size 2, $c={\left[c_{x}\;c_{y}\right]}^{⊤}$ denotes the position of the COM, $\zeta ={\left[\zeta_{x}\;\zeta_{y}\right]}^{⊤}$ is the DCM, $p={\left[p_{x}\;p_{y}\right]}^{⊤}$ represents the position of the ZMP and **Ω** = [***ω ω***]^*⊤*^. This system shows that the COM is always converging to the DCM, and controlling the DCM is enough to develop stable locomotion. Thus, the DCM tracker can be formulated as:

$$u_{\zeta }=-K_{\zeta }e_{\zeta },$$ (4)

where ***K***_*ζ*_ represents the controller gains, $e_{\zeta }={\left[\zeta_{d}-\zeta ,\;{\dot{\zeta }}_{d}-\dot{\zeta }\right]}^{⊤}$ , $\zeta_{d},\dot{\zeta_{d}}$ are the desired DCM and its time derivative, which are generated by the DCM planner (see Figure 2).

## 3 Learning residual

Although the gait generator produces stable locomotion, it does not generalise well to unforeseen circumstances. This section presents our developed learning framework that can learn *residual actions* on top of the kernel pattern generator. The objective is to regulate control parameters such as the COM height and stride time, and also learn model-free skills to generate compensatory joint actions.

### 3.1 Baseline

The Proximal Policy Optimisation (PPO) algorithm Schulman et al. (2017) is selected as the baseline RL algorithm due to its computational efficiency and good performance in high-dimensional environments. PPO (Schulman et al., 2017) is an actor-critic algorithm that uses a clipping function to constrain the policy update directly inside the objective function, thus preventing it from being too greedy. PPO seeks to balance the trade-off between exploration and exploitation by iteratively improving the policy while simultaneously updating an estimate of the value function. This learning problem can be formally described as a Markov Decision Process (MDP)—a tuple $\left\langle S,A,\Psi ,p,r\right\rangle$ , where *S* is the set of states, *A* is the set of actions, Ψ ⊆ *S* × *A* is the set of admissible state-action pairs, *p*(*s*, *a*, *s*′): Ψ × *S* → [0, 1] is the transition function, and *r*(*s*, *a*): Ψ → I R is the reward function. The PPO algorithm is formulated as an optimization problem, where the objective is to maximize the expected return of the policy *r*(*s*, *a*). This is achieved by iteratively updating the policy parameters using a clipped surrogate objective function that limits the change in the policy at each update step. The surrogate objective function involves two terms: the ratio of the new and old policies multiplied by the advantage estimate, and a clipped version of the ratio that restricts the magnitude of the change in the policy.

### 3.2 Data augmentation with exploiting the symmetry

We intent to extend this algorithm with symmetric data augmentation based on static domain knowledge as most of humanoid robots have reflection symmetry in the sagittal plane, which can be leveraged to reduce the learning time and guide the optimisation algorithm in creating a human-like behaviour. In order to reduce the mathematical model by exploiting its redundancy and symmetry, Ravindran and Barto (2001) proposed the MDP homomorphism formalism, which describes a transformation that simplifies equivalent states and actions. Let *h* be an MDP homomorphism from $M=\left\langle S,A,\Psi ,p,r\right\rangle$ to $M^{\prime }=\left\langle S^{\prime },A^{\prime },\Psi^{\prime },p^{\prime },r^{\prime }\right\rangle$ , and *A*_*s*_ be the set of admissible actions in state *s*. The concept of MDP symmetries is a special case of this framework where *f*: *S* → *S*′ and $g_{s}:A_{s}\to A_{f\left(s\right)}^{\prime }$ are bijective functions. An MDP isomorphism from and to the same MDP can be considered an automorphism that satisfies:

$$\begin{array}{cc} p\left(f\left(s\right),g_{s}\left(a\right),f\left(s^{\prime }\right)\right) & =p\left(s,a,s^{\prime }\right),\;\forall s,s^{\prime }\in S,a\in A_{s},\\ and\;r\left(f\left(s\right),g_{s}\left(a\right)\right) & =r\left(s,a\right),\;\forall s\in S,a\in A_{s}. \end{array}$$ (5)

After performing a grid search, the batch size was set to 8192 samples and the learning rate to 3e − 4 (using a linear scheduler). For each episode, an MDP trajectory *j* is characterised by a sequence of states, actions and rewards such that *j* = {*S*_0_, *A*_0_, *R*_0_, *S*_1_, *A*_1_, *R*_1_, …}. Each trajectory is used to produce a set of samples *k* = {{*S*_0_, *A*_0_, *Ad*_0_, *V*_0_}, {*S*_1_, *A*_1_, *Ad*_1_, *V*_1_}, …}, where *V*_*i*_ is obtained from the *λ*-return as defined by Sutton and Barto (Sutton and Barto, 2018), and serves as value target for the update function; and *Ad*_*i*_ is the generalised advantage estimate (Schulman et al., 2018).

Our proposal is to partially augment data by copying and transforming a fraction of the acquired samples. Different augmentation ratios are tested in Section 5. As an example, consider the addition of symmetrical samples with a ratio of 50%. Following 5), each batch of samples is artificially built as {*W*_1_, *W*_2_, *u*(*W*_2_), *W*_3_, *W*_4_, *u*(*W*_4_), …} where *u*(*W*_*i*_) = {*f*(*S*_*i*_), *g*_*s*_(*A*_*i*_), *Ad*_*i*_, *V*_*i*_}. The observations’ normalisation is continuously updated by calculating the mean and standard deviation of each observation. However, both of these metrics are shared among the two symmetric groups to ensure that no asymmetrical bias is introduced.

### 3.3 Network architecture

**FIGURE 3:**
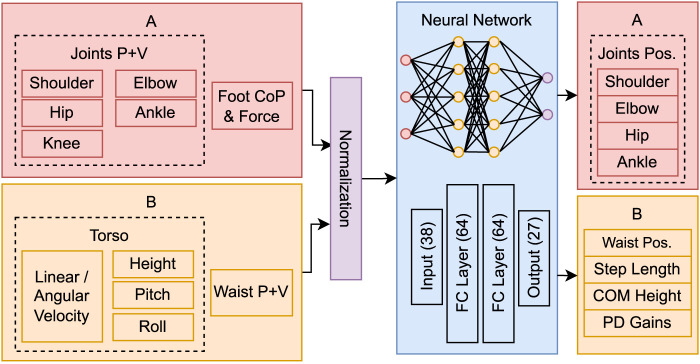
Network architecture, system space parameters and symmetry transformation groups used for data augmentation: **(A)** reflection symmetry transformation and **(B)** no transformation or inversion.

The network architecture and system space parameters are depicted in Figure 3. The observations comprise the position of 6 joints: shoulder, hip and waist with 3 degrees of freedom (DoF), ankle with 2 DoF, knee and elbow with 1 DoF. All joints are mirrored except the waist. Additional observations include the foot relative centre of pressure (in *x* and *y*) and respective force magnitude, the torso’s linear and angular velocity, height, pitch, and roll; totalling 38 state variables. This data is fed to a neural network with 2 hidden layers of 64 neurons, that produces joint residuals, which are added to the precomputed trajectories; and high-level parameters to regulate the kernel pattern generator: step length, COM height, and two PD gain vectors (***K***_Φ_ from (Eq. 2) and ***K***_*ζ*_ from (Eq. 4).

The system space parameters are grouped into two symmetry transformations categories for data augmentation. Category A includes duplicated observations that are mirrored, considering the sagittal plane. Category B includes unique observations that may remain unchanged (e.g., torso’s height) or suffer an inversion transformation (e.g., roll angle).

### 3.4 Reward function

The reward function tries to achieve one fundamental goal of balancing while keeping cyclic movement patterns. The balance goal seeks to keep the robot on its feet in all situations. The subgoal of performing cyclic movement patterns has the purpose of improving the human-like aspect of the behaviour. Specifically, it tries to reduce the neural network’s influence (NNI) when there is no need to intervene. Both of these notions can be expressed through the following reward:

$$R=1-\overset{\mathrm{NNI}}{\overbrace{\frac{1}{J}\overset{J}{\underset{i}{\sum }}\frac{\left|\delta_{i}\right|}{S_{i}}}},$$ (6)

where *δ*_*i*_ is the residual applied to joint position *i*, *J* is the number of joints, and *S*_*i*_ is the residual saturation value (±0.25*rad*). It is important to note that the NNI component’s goal is not to reduce energy consumption or range of motion, since it is only applied to the residuals and not the hybrid controller’s output. According to this reward function, the robot aims to maximize the accumulated rewards in each episode. The episode will be ended as soon as the robot loses its balance and falls down. This means that the robot receives a reward for maintaining its stability and is penalized for any deviation from this stability (as measured by the residual; the more residual, the less reward). Through this reward function, we have established a clear criterion for determining when the robot’s stability is compromised.

## 4 Simulation scenarios

To validate the performance of the proposed framework, a set of two learning scenarios and one test scenario has been designed. The goal of this structure is to prepare the physical robot to handle real-world adverse conditions. We use the COMAN robot in PyBullet (Coumans and Bai, 2016)—an environment based on the open source Bullet Physics Engine which is a highly capable open-source physics engine and simulator that is designed to facilitate research and development in the fields of robotics, machine learning, and computer graphics. It provides a versatile and powerful platform for simulating complex systems with a high degree of accuracy and speed, and supports a wide range of features, including collision detection, contact dynamics, and rigid and soft body dynamics. The simulated robot is 95 cm tall, weighs 31 kg, and has 23 joints (6 per leg, 4 per arm and 3 between the hip and the torso). In our simulations, we have developed position controllers to control the actuators whose parameters including the maximum torques are set according to the robot specs presented in Tsagarakis et al. (2013) (peak torque of 55 Nm for all leg joints). Also, the stiffness for the ankle, knee and hip joints (ka, kk, kh) are different and have been tuned using the method presented in Tsagarakis et al. (2013). In our simulation, the stiffness set (ka, kk, kh) = (0.300, 0.241, 0.195) Nm/rad. By tuning these gains, we make sure that the controller was able to track the desired setpoint and achieve the desired performance.

### 4.1 Learning scenario: Flat terrain

**FIGURE 4:**
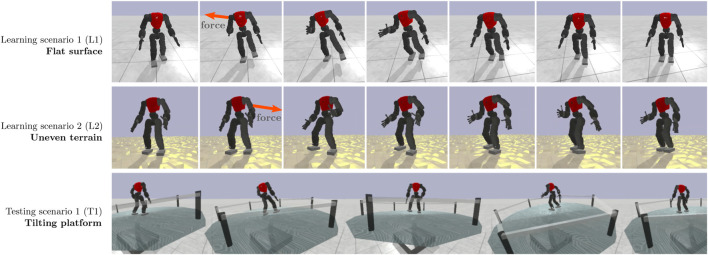
Illustration of learning scenarios: (1) a flat surface where the robot learns how to recover from external forces (L1); (2) an uneven terrain with vertical heights of 2 cm (L2); (3) Unseen scenarios on a tilting platform that moves erratically (T1).

The first learning scenario (L1) is composed of a flat platform (see Figure 4, top row), where the robot is initially placed in a neutral pose. It then starts to walk in place, while being pushed by an external force at random intervals, between 2.5 and 3.0 s. The force is applied for 25 ms and ranges from 500 N to 850 N (impulse 12.5–20 N s). Its point of application is fixed at the torso’s centre and its direction is determined randomly in the horizontal plane. The robot’s objective is to avoid falling. The episode ends when the robot’s height drops below 0.35 m.

### 4.2 Learning scenario: Uneven terrain

The second learning scenario (L2) is an extension of the first one, where the flat surface is replaced by an uneven terrain with perturbations that can reach 0.02 m, as depicted in Figure 4, middle row. The external force dynamics are the same.

### 4.3 Testing scenario: Tilting platform

The testing scenario (T1) was designed to evaluate the generalisation capabilities of the hybrid controller in unexpected circumstances. It is characterised by a tilting cylindrical platform (see Figure 4, bottom row), which is supported by two actuators that move on the *x* and *y*-axes, and range between −15 deg and 15 deg. The position of each actuator is given by adding a random component *r* ∈ [−8°, 8°] to a correcting component *c* = 0.35 × *P*, where *P* is the position of the robot in the opposite axis to the actuator. The goal of the latter component is to keep the robot on top of the platform by encouraging it to move to the centre. The episode starts in a neutral state with the robot walking in place, and it ends when the robot falls, as in previous scenarios.

## 5 Simulations

This section is focused on a set of assessments and analysis of the proposed framework regarding overall performance, optimised policy behaviour, symmetry, robustness, and applicability to walking.

### 5.1 Baseline and overall performance analysis

As it is detailed in Section 2, the gait generation kernel is designed based on the DCM concept, which is the state-of-the-art method for developing a walking engine using conventional control methods. As our baseline, we use this kernel without adding residuals or modulating its parameters. To assess the performance of the baseline, we examine the maximum impulse that the baseline can withstand while walking in place on a flat terrain. To do so, similar to the scenario L1, while the robot is walking in place, it is subjected to an external force at random intervals, between 2.5 and 3.0 s, with a fixed impact duration of 25 ms and fixed point of application at the torso’s centre and its direction is determined randomly in the horizontal plane. The force amplitudes start at 100 N and will be increased by 10 N after 10 successful recoveries in a row. Simulation results showed that 380 N (impulse 9.5 N s) was the maximum force that the robot could resist.

Furthermore, seven policies were trained in scenario L1 to evaluate the effectiveness of (Eq. 1) adding the residuals to the arms, (Eq. 2) adding the residuals to the legs, (Eq. 3) adding the residuals to the arms and the legs, (Eq. 4) modulating the kernel parameters without adding residuals, (Eq. 5) modulating the kernel parameters and adding residuals to the arms, (Eq. 6) modulating the kernel parameters and adding residuals to the leg and (Eq. 7) modulating the kernel parameters and adding residuals to the arms and legs. All optimisations ran for 50 M (million iterations). Then, the baseline test scenario has been repeated to assess the performance of the policies. The results are summarized in Table 1. The results showed that adding residuals to the arms and legs improves the withstanding level of the robot up to 34.2% and it reaches 42.1% just by modulating the kernel parameters. Still, it goes up to 118.1% while using both alongside each other. Bold values represent adding residuals and modulating the kernel parameters are two important factors that can improve the stability impressively.

**TABLE 1:** Maximum disturbance rejection using different combinatorial use of gait modulation and compensatory actions.

Configuration	Maximum force (N)	Maximum impact (N s)
Baseline	380	9.5
Adding arms residuals	400	10
Adding legs residuals	430	10.75
Adding arms and legs residuals	510	12.75
Modulating kernel parameters	540	13.5
Modulating kernel param. and		
adding arms residuals	580	14.5
Modulating kernel param. and		
adding legs residuals	790	19.75
**Modulating kernel param. and adding arms and legs residuals**	**830**	**21**

Bold values represents the best combination and results.

### 5.2 Performance analysis of symmetrical policies

**FIGURE 5:**
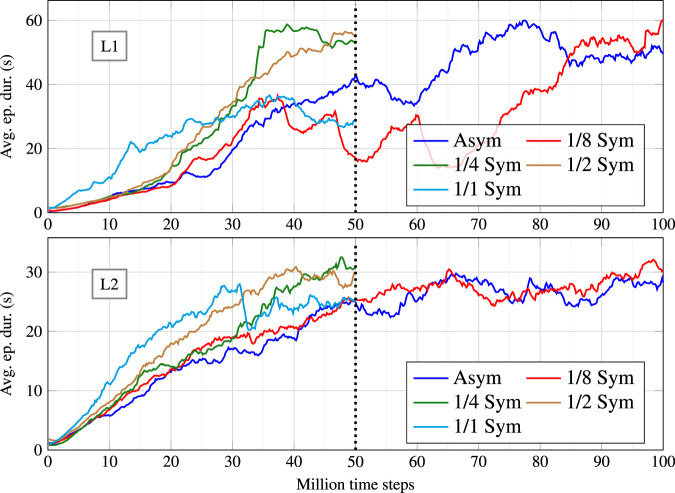
Learning curves for the policies trained in scenario L1 (top) and L2 (bottom), under different symmetry configurations.

Five different symmetry ratios were tested per learning scenario, totalling ten different configurations. The symmetry ratios were 0 (no data augmentation), 1/8 (1 symmetrical sample is generated per 8 acquired samples), 1/4, 1/2 and 1/1 (full symmetry). For each configuration, five policies were trained. Figure 5 depicts the learning curves for the best policy in each configuration. The results are grouped according to the training scenario (L1 above and L2 below). Most optimisations ran for 50 M time steps. However, the asymmetric and 1/8 symmetry configurations needed 100 M time steps to reach a plateau. For the configurations that included data augmentation, the best performing ratios were 1/4 and 1/2, with similar results. In a subjective visual evaluation, the 1/2 ratio policy seems to be marginally better in producing a human-like behaviour. For the remainder of this section, we will compare in greater detail the asymmetric version with the 1/2 symmetric version. A video including the results is attached.

It is important to note that the average episode duration reported by these learning curves results from a stochastic policy with a non-negligible random component. To better assess the optimised policies, they were tested in each scenario (including T1 — the only test scenario) for 1000 episodes using the corresponding deterministic policy. Moreover, to be fair with every approach, only the evolution until 50 M time steps was considered in these tests. Although the 1/8 symmetric version on L1 presents an atypical evolution, it was chosen because it achieved the best performance among concurrent policies. Table 2 compares the average performance of 4 policies against the baseline. The first four columns indicate, in this order, the episode duration, in seconds, in scenario L1, L2 and T1; and the neural network influence (examined later in this section).

**TABLE 2:** Statistical average duration of resisting random force perturbations (500 N–850 N, impulse 12.5–20 N s) in different learning configurations.

Learning configuration	Episode duration (s)			N. Network influence	M. Sym. Index
	L1	L2	T1		
Baseline	3.47	1.51	1.87	—	—
L1 Asym	104.5	5.1	4.8	0.072	1.42
L1 1/2 Sym	202.2	4.6	4.8	0.055	1.19
**L2 Asym**	**321.9**	**34.2**	**27.8**	**0.165**	**1.23**
**L2 1/2 Sym**	**193.7**	**43.5**	**21.0**	**0.127**	**0.99**

Bold values represents the best combination and results.

The baseline version (without residuals) is not able to handle the strong external forces applied in scenario L1, falling on average after 3.47 s, which is typically after the first push. On L2, it falls almost immediately due to the floor perturbations, an outcome which is also seen in T1. All four learned policies are a great improvement over the baseline. As expected, the last two policies that learned on L2 were able to generalise successfully when tested on L1 or T1, and, on the opposite side, the policies that learned on L1 did not perform well in unforeseen circumstances.

However, some interesting results were not expected. During training, the symmetrically-enhanced policies performed better but, while testing in distinct scenarios, the asymmetrical policies generalised better. Another interesting result is that the asymmetrical L1 policy performed worse in its own scenario (104.5 s) than the asymmetrical L2 policy (321.9 s).

An initial hypothesis to explain this outcome would be to assume that learning on uneven terrain requires additional effort to maintain balance and, consequently, produces a better policy. In fact, considering that the robot is already pushed periodically, gravity acts as an additional external force when the robot is standing on a slope. On its own, this explanation is insufficient because the robot that learned on the flat surface could continue the optimisation process until it found a better policy. However, this would only be true if the reward was solely focused on raw performance.

To understand this result further, we analysed the NNI column of Table 2, whose metric is defined in (Eq. 6). Since L2 and L2 Sym require additional effort to counteract gravity when standing on a slope, the robot learned to sacrifice its immediate reward by applying larger residuals in order to avoid falling. Naturally, this is a trade-off between cyclic movement patterns and raw performance. Moreover, learning an asymmetrical behaviour can arguably be considered more complex, leading to a higher network influence, which may explain why it generalises better than the symmetrical policies.

### 5.3 Optimised policy behaviour analysis

**FIGURE 6:**
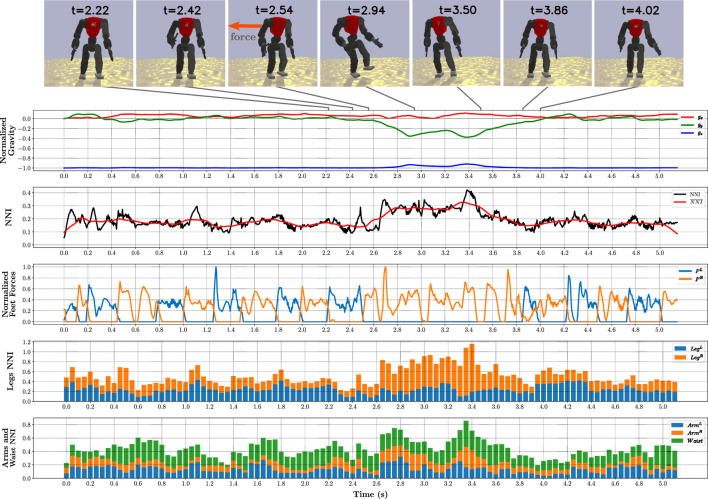
Analysis of simulation using the asymmetrical L2 policy on the L2 scenario for 5 s, with a single external push, applied to the robot’s torso for 0.025 s with a force of 850 N. Seven snapshots before and after the push are presented. During entire simulation period, different metrics were sampled at 200 Hz as: normalised gravity vector, relative to the robot’s torso; global NNI, as defined in (Eq. 6); normalised feet forces; and NNI per joint group.

To present more detail about the overall behaviour of the optimised policies and to explain how they improve the robot’s stability significantly, we selected the asymmetrical L2 policy to represent all the optimised policies and tested it on the L2 scenario for 5 seconds while recording all observations and actions (200 Hz). In this simulation, while the robot was walking in place, at t = 2.54s, it was subjected to a 850 N external push at its torso’s centre for 0.025s. The robot was able to counteract this force and regain its stability. A set of snapshots along with five important plots are depicted in Figure 6, including the normalised gravity vector and feet forces, and the NNI on different joint groups.

The first plot shows the normalised gravity vector, relative to the robot’s torso. After applying the push, the robot leans considerably, with an inclination of 23°, which can be characterised as a severe perturbation. Before the push, the average NNI $\left(\bar{\text{NNI}}\right)$ is less than 0.2. The robot applies small corrections to keep its stability while walking in place on the uneven terrain. After triggering the external push, the network’s influence rises 50%, which translates into larger residuals, as a response to regain stability. After returning to a normal state, the NNI is smoothly reduced. These results validate the policy’s objective stated in (Eq. 6), by adjusting the NNI according to the robot’s requirements at a given moment.

To identify the distinct strategies at play, we broke down the network’s influence into groups of limbs and waist, and chose feet forces as an additional metric. The total force acting perpendicular on each foot encodes the actual stride time and, by inspecting its plot, we can infer that even before the push, changing the stride time is one of the employed strategies.

The network’s influence per group was obtained by applying the NNI formula from (Eq. 6) to the joints in a given group. The 2 bottom plots represent the groups of joints associated with both legs, both arms, and waist, using a stacked bar chart, where each bar represents the mean of 10 control steps (0.05 s).

During the push, the support leg had the most expressive response in comparison with the other joint groups. This behaviour is best understood by analysing a slow-motion video of the push (available as Supplementary Video S1). The robot starts hopping on the support leg, while using its upper body as a complementary strategy to shift the COM by swinging the arms as required. This process persists until the robot is stable enough to return to the initial walking gait. These strategies, along with adjusting the stride time and COM height, allow the robot to perform seamless transitions, like humans would do unconsciously.

### 5.4 Symmetry analysis

Symmetry is an important property of human behaviours, often associated with positive reactions, as opposed to asymmetry (Evans et al., 2012). However, humans are not perfectly symmetrical, and unbalanced gait patterns can be perceived as unimpaired or normal, within reason (Handžić and Reed, 2015). Therefore, in the context of human-like behaviours, the symmetry of a policy should be leveraged, but not to the point where it becomes a hard constraint. In these simulations, the kernel pattern generator produces symmetrical trajectories upon which the neural network residuals are applied. To evaluate the residuals symmetry, we built upon the concept of Symmetry Index (SI) proposed by Robinson et al. (1987). The original method compares the kinematic properties of each lower limb. To address the issues caused by abstracting the kinematic properties of each joint, we propose the Mirrored Symmetry Index (MSI):

$$MSI=\frac{\|\delta_{t}-\delta_{t}^{\prime }{\|}_{1}}{0.5\times \left(\|\delta_{t}{\|}_{1}+\|\delta_{t}^{\prime }{\|}_{1}\right)},$$ (7)

where $\delta_{t}=\left[\delta_{1}^{t},\ldots ,\delta_{n}^{t}\right]$ is the vector of residuals applied to each joint during time step *t*, ‖ ⋅‖_1_ is the *ℓ*1-norm, and $\delta_{t}^{\prime }$ is the vector of residuals applied to the symmetric set of joints if the current state was also symmetrically transformed, i.e., $\delta_{t}^{\prime }∼\pi \left(\cdot |f\left(S_{t}\right)\right)$ , where *π* is a stochastic policy. Instead of evaluating an average kinematic feature, the MSI computes a symmetry index at each instant, which can then be averaged for a full trajectory to obtain a global symmetry assessment.

As seen in Table 2, the policies which were learned using the data augmentation method obtained a lower MSI value, when compared to the other two policies. The results do not show a large reduction, which can be explained by the analytical controller’s role in regulating the trajectory symmetry, and the relaxed data augmentation restriction imposed to the network.

**FIGURE 7:**
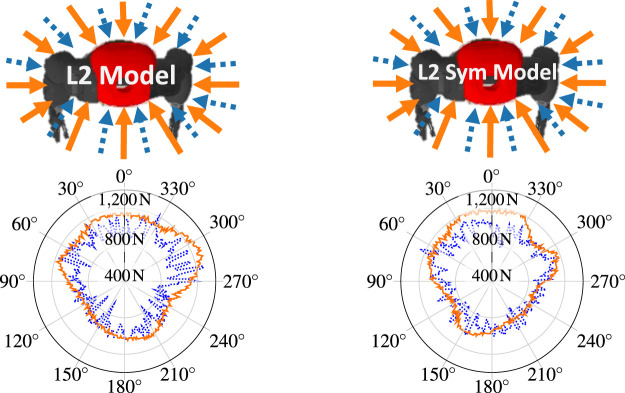
Maximum radially applied external force from which the robot can consistently recover as a function of the direction of application, where zero degrees corresponds to the front of the robot. On the left is shown the policy which learned on L2 and on the right L2 Sym. The force was applied both in the flat terrain (solid orange line) and the uneven terrain (dotted blue line). The radial y-axis range is [400,1400]N. The maximum withstood force was 1300 N for the L2 policy in the flat terrain, at 290°.

To assess the notion of symmetry on a practical scenario, the policies trained on L2 and L2 Sym were subjected to a test where an external force with constantly increasing norm is radially applied to the robot in a given direction. When the robot is no longer able to recover consistently (more than 50% of the trials), the maximum force is registered and another direction is tested. The result can be seen in Figure 7 on the flat terrain (solid orange line) and uneven terrain (dotted blue line). In both cases, the robot is able to better withstand forces that are applied to the front (around 0 deg). On one side, the symmetrically-enhanced version presents a more balanced result, which can be visually perceived. On the other side, the asymmetrical policy can withstand larger forces around 300 deg. This difference consists of a trade-off between symmetry and raw performance.

### 5.5 Robustness

**FIGURE 8:**
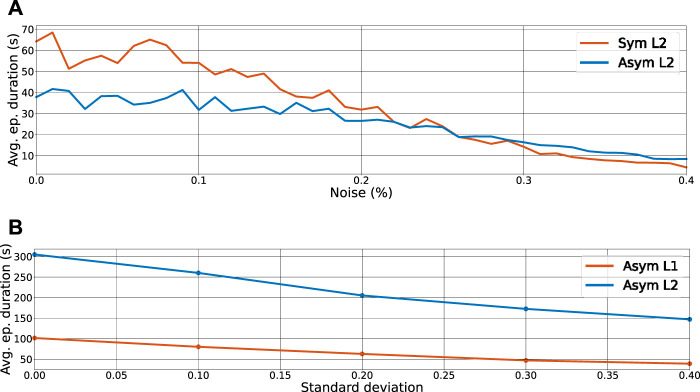
Robustness with regarding to the measurement noise and model uncertainties. **(A)** Average episode duration as a function of noise applied to the state observations for the symmetrical (orange line) and asymmetrical (blue line) policies learned and tested on the uneven terrain. **(B)** Average episode duration as a function of standard deviations of mass uncertainties applied to the masses from a normal distribution N(0,σ) .

Robustness with regarding to measurement noise and model uncertainties are matter of significant concern on real applications and feasibility of transferring the trained policies on real robots. To assess the robustness with regarding to measurement noise, the state variables are multiplied by a random factor that follows a uniform distribution z∼U(1.0,N) where *N* ranges from 1.0 to 1.4, i.e., 0%–40% of maximum noise. Figure 8A shows the average impact of this artificial perturbation on the average episode duration, on the uneven terrain scenario, while being pushed by an external force (described in Section 4.1) with a fixed interval of 3.5 s. Both the symmetrical and asymmetrical policies can withstand a maximum noise of 20% without dropping below the 30 s mark, which attests the policies’ robustness in considerably noisy scenarios.

Model uncertainties, which are primarily caused by mass inaccuracies, inertia, link dimensions, communication delay, and disturbances, prevent a straight transfer of trained policy from simulation to reality. To evaluate the performance of the trained policies in terms of model uncertainties, we selected the asymmetrical L1 and asymmetrical L2 policies and tested them on the scenario L1 while adding uncertainties of masses from a normal distribution with different standard deviations N(0,σ) . The averaged results for 50 episodes are depicted in Figure 8B. As the results showed, although mass inaccuracies affect performance, both policies can tolerate mass inaccuracies up to 20% without dropping below 50%.

### 5.6 Generalization to walking

**FIGURE 9:**
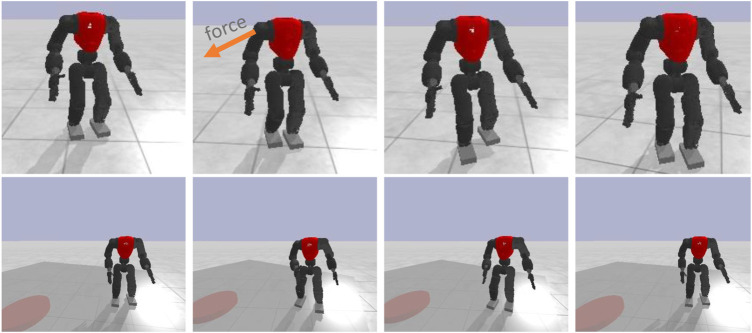
Trained neural network policies tested in other gaits: walking forward and walking in place on a rotating platform.

As the presented results in Section 5.2 showed, the trained policies capable of generalizing its knowledge in the unseen test scenario (T1). To investigate more this capability, the policies trained in this work were applied to different gaits managing to attain a satisfactory performance while walking forward and being pushed, and while walking in place on a rotating platform (see Figure 9). Changing direction or walking sideways can cause instability with the current configuration. However, these results, as shown in the accompanying video, reveal a significant generalisation ability, considering that this new task was not trained specifically during the learning process.

## 6 Conclusion

In this paper, we proposed a locomotion framework based on a tight coupling between analytical control and deep reinforcement learning to combine the potential of both approaches. First, we developed a closed-loop fully parametric gait generation kernel. Then, we designed a learning framework which extends PPO with symmetric partial data augmentation to learn residuals actions. This hybrid approach aims at unlocking the full potential of the robot by exploiting the consistency of the analytical solution, the generalisation ability of neural networks, and the policy’s symmetry, while not totally constraining the exploration of asymmetric reactions. A set of policies were trained to evaluate the effectiveness of adding residuals and modulating the kernel parameters. The results validated that employing both techniques alongside each other can improve the performance up to 118.1%.

We provided robustness analysis to different uncertainties and disturbances including measurement noise and model uncertainties, which are primarily caused by mass inaccuracies, inertia, link dimensions, communication delay, and disturbances. The results showed that the trained policies in the simulation are robust in the presence of noise and model inaccuracies, and have the feasibility of future deployment on real robots. The further cap of transferring the algorithm to the real hardware has not yet studied in this paper.

The symmetry enhanced policies were able to perform better in the scenarios where they learned, and with less samples, but were not able to generalise as well in unforeseen circumstances. However, the difference is partially explained that the reward function’s penalty is less restrictive in challenging conditions. Generalisation capabilities of the proposed framework has been evaluated through a set of simulation scenarios. The results showed that the policies trained in this work can generalise to other gaits, such as walking forward and walking in place on a rotating platform. Future work can explore the application of this hybrid approach to other types of gaits, such as running and climbing.

## Data Availability

The raw data supporting the conclusion of this article will be made available by the authors, without undue reservation.
